# Enhancing starch properties through dual modification: Ultrasonication and acetic acid treatment of non-conventional starches

**DOI:** 10.1016/j.ultsonch.2025.107301

**Published:** 2025-03-04

**Authors:** Likhitha Yadav Prakruthi, Hari Krishnan, Tamma Medha, K. Kumarakuru, P. Vasantha Kumari, Challa Surekha, Hemasundar Alavilli, Deepika Kaushik, Abeer Hashem, Nouf H. Alotaibi, Graciela Dolores Avila-Quezada, Elsayed Fathi Abd_Allah, Mukul Kumar, Chagam Koteswara Reddy

**Affiliations:** a Department of Lifesciences, GITAM (Deemed to be University), Visakhapatnam 530045, India; b Center of Multidisciplinary Unit of Research on Translational Initiatives (MURTI), GITAM (Deemed to be University), Visakhapatnam 530045, India; c Department of Food Processing Technology, PSG College of Arts and Science, Coimbatore 641014, India; d Department of Food Science and Nutrition, Mount Carmel College, Bangalore, India; e Department of Biotechnology, Faculty of Applied Sciences and Biotechnology, Shoolini University, Solan 173229, India; f Botany and Microbiology Department, College of Science, King Saud University, P.O. Box. 2460, Riyadh 11451, Saudi Arabia; g Chemistry Department, College of Science, King Saud University, P.O. Box. 2460, Riyadh 11451, Saudi Arabia; h Facultad de Ciencias Agrotecnológicas, Universidad Autónoma de Chihuahua, 31350, Chihuahua, Chihuahua, Mexico; i Plant Production Department, College of Food and Agricultural Sciences, King Saud University, P.O. Box. 2460, Riyadh 11451, Saudi Arabia; j Department of Food Technology and Nutrition, Lovely Professional University, Phagwara, Punjab 144411, India

**Keywords:** Non-conventional starches, Ultrasonication, Acetic acid modification, Dual modification

## Abstract

This study investigated the effects of ultrasonication (US) and acetic acid treatments on starches extracted from non-conventional sources: elephant foot yam (NES), cassava (NCS) and sweet potato (NSP). The starches underwent ultrasonication at 40°C for 3, 9, and 15 min, followed by acetylation, with native starches used as control. The morphological, physicochemical, and functional properties were comprehensively analyzed. Results revealed that increased treatment time significantly (*p*<0.05) affected the starches functional properties, morphology and crystallinity. Amylose content was highest in NES (22.85 %), followed by NSP (21.05 %) and NCS (19.28 %). Following dual modification, a significant reduction in amylose content was observed in ultrasonic-assisted acetylated starches. Morphological analysis revealed granular aggregation, surface changes, and the formation of pores and cracks. X-ray diffraction (XRD) patterns demonstrated that NSP and NES starches exhibited major peaks characteristic of C-type starches, while NCS starches displayed an A-type pattern. Following ultrasound-assisted acetylation, the crystalline structures of all starches remained largely unchanged, although relative crystallinity slightly decreased compared to native starches. The oil absorption capacity and tap density of NES increased with dual modification, suggesting enhanced hydrophobicity. These findings highlight the potential of dual modification to improve starch properties for industrial applications, including confectionery, edible films, tablet binders, and encapsulation.

## Graphical abstract

**Figure:**
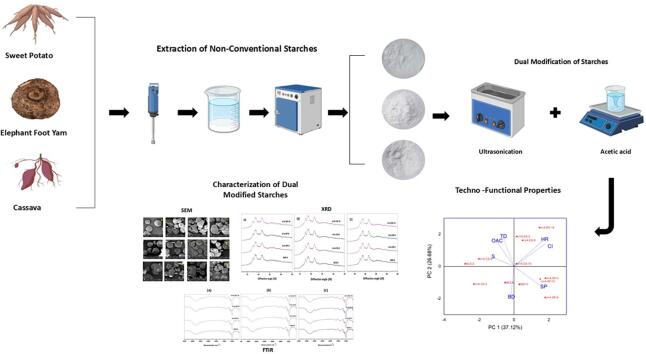

## 1 Introduction

Starch, a semi-crystalline and eco-friendly polymer, is a naturally abundant biopolymer found in cereals, grains and tubers. Due to its versatility, starch is widely used in food industries for functions such as thickening, stabilizing, texturing, gelation, encapsulation, and extending shelf life, all of which significantly enhance the quality and texture of food products [1], [2]. The functional properties of starch are attributed to its composition, mainly amylose and amylopectin, which enable its diverse applications [3]. Despite its widespread use, native starch has several limitations, such as high retrogradation, poor paste clarity, and susceptibility to extreme temperature and pH conditions [4]. As a result, extensive research has focused on understanding the structure, physicochemical properties, and isolation of starches from different sources to overcome these limitations and optimize their functional properties.

In recent years, various modification techniques—physical, chemical, enzymatic, and hybrid methods—have been explored to enhance the techno-functional properties of starches [3]. These modifications aim to improve properties such as film-forming ability, adhesiveness, and resistance to retrogradation [5]. Among the numerous starch sources, elephant foot yam, cassava, and sweet potato stand out due to their unique properties and nutritional benefits. Elephant foot yam, a tuber from the *Araceae* family, is particularly rich in carbohydrate content, along with proteins, fibers and essential minerals [4]. It contains 10–30% amylose and 70–90% amylopectin, which contribute to its potential health benefits [6]. However, elephant foot yam starch is prone to high moisture content, which limits its shelf life despite its valuable resistant starch content [7]. Similarly, cassava starch, a key commercial crop in tropical regions, is prized for its high yield, affordability, and ease of extraction [8]. However, the practical application of native starch is limited due to its low solubility, high paste viscosity, and retrogradation [1]. Sweet potato starch, while rich in dry matter (80%), is also widely used in food and bioethanol production, but exhibits low solubility, and high retrogradation [9].

Recent advancements in chemical modifications, such as acetylation, offer promising solutions to overcome the limitations of native starches [10]. Acetylation improves the interaction of starch with water and oil, enhancing properties like gel solubility, swelling power, and viscosity, while lowering the initial gelatinization temperature [11]. Traditional acetylation methods have utilized acetic anhydride or alkaline reagents, but alternative approaches using acetic acid or organic acids have also been explored. However, single modification techniques may not always yield optimal results, prompting interest in dual modification strategies. Combining chemical modifications with other techniques has proven to enhance starch functionality more effectively than standalone methods.

Ultrasonication, known for its environmental advantages and operational ease, has emerged as an effective technique for improving starch properties [12]. By enhancing the surface area of starch granules through cavitation and shear forces, ultrasonication accelerates chemical reactions and improves yield. It also reduces partially gelatinized starch particles, improving moisture permeability, transparency, and surface uniformity [13]. Previous studies have shown that combining ultrasonication with citric acid treatment can significantly improve the functionality of starches, expanding their applications in bakery products and as thickeners [14]. The dual modification of starches offers the potential to achieve specific characteristics that cannot be attained through single-modification methods, thus opening new avenues for enhancing the functionality of starches from non-conventional sources.

This study aims to investigate the dual modification of starches isolated from non-conventional sources—elephant foot yam, cassava, and sweet potato—through ultrasonication and acetylation. The morphological, physicochemical, and functional characteristics of these modified starches were analyzed. The findings from this research are expected to provide valuable insights into selecting the most suitable starch sources for various industrial applications, including edible films, coatings, and encapsulation.

## 2 Materials and methods

### 2.1 Materials

Root tubers of cassava, elephant foot yam and sweet potato were procured from the local market in Visakhapatnam, India. Analytical grade chemicals and reagents were obtained from Hi-Media Co. (Mumbai, India).

### 2.2 Starch extraction

The starch extraction was performed using the method described by Medha et al. [12]. The tubers were first peeled and cut into small pieces, which were immersed in 0.1% (w/v) sodium metabisulfite solution. The samples were homogenized in a blender and suspended in a 4% NaCl solution. The resulting slurry was filtered through a 100 μm sieve, and the filtrate was centrifuged at 3000 rpm for 15 min. This procedure was repeated four times to ensure maximized starch recovery. The collected white starch was then oven-dried at 45°C for 24h, powdered, and stored at room temperature in a sealed container.

### 2.3 Proximate composition and amylose content determination of starch

The proximate composition of the isolated starches, including moisture content, ash, crude fat and crude protein levels, was determined according to the methods outlined by Reddy et al. [6]. The amylose content and total starch content were measured using the iodine-binding method, as described by Medha et al. [12].

### 2.4 Preparation of dual-modified starches

Dual modification of the isolated starches from cassava (NCS), elephant foot yam (NES) and sweet potato (NSP) was carried out using a combination of ultrasonication and acetylation as detailed below.

#### 2.4.1 Ultrasonication

Starch slurries (25% w/v) were subjected to ultrasonication in a bath-type ultrasonic processor (300W) at 40°C for different durations (3, 9 and 15 min). Following ultrasonication, the slurries were centrifuged at 5000 rpm for 15 min at room temperature. The starch sediment was collected and dried in a hot air oven at 45°C until the moisture content reached ∼10%, and subsequently powdered and sieved for further analysis.

#### 2.4.2 Acetylation

The starch slurry was mixed with acetic acid in a 1:4 (w/v) ratio and continuously stirred magnetically, followed by incubation at 45°C for 2h. After incubation, the mixture was centrifuged at 5000 rpm for 15 min. The acetylated starch sediment was collected, dried in a hot air oven, and stored at room temperature for subsequent analysis. The following codes were assigned to the samples: NSP-0 (native sweet potato starch), U+A SP-3, U+A SP-9, U+A SP-15 (ultrasonic-assisted acetylated NSP starches); NES-0 (native elephant foot yam starch), U+A ES-3, U+A ES-9, U+A ES-15 (ultrasonic-assisted acetylated NES starches); and NCS-0 (native cassava starch), U+A CS-3, U+A 112 CS-9, U+A CS-15 (ultrasonic-assisted acetylated NCS starches). These codes represent native starches and ultrasound-acetylated modified starches subjected to treatment times of 3, 9, and 15 min, respectively.

### 2.5 Morphological characteristics

The morphology of native and modified starches was examined using a field-emission scanning electron microscope (FE-SEM, Tescan Mira 3, Czech Republic) [1]. Samples were mounted on aluminum stubs using conductive double-sided tape, coated with gold, and analyzed at an acceleration voltage of 5 kV.

### 2.6 X-ray diffraction

X-ray diffraction (XRD) analysis was conducted using a Bruker D8 Advance, Germany equipped with CuKα radiation (1.5406 Å) at 40 mA and 40 kV with a scanning rate of 1.5°/min across a scattering range of 2*θ* from 10° to 50° and the relative crystallinity was calculated, following the procedure described by Reddy et al. [11].

### 2.7 Functional group analysis

Fourier transform infrared (FTIR) spectra of both native and dual-modified starches were recorded using an FTIR spectrometer (ALPHA II, Bruker, Germany). The spectra were obtained in the wavelength range of 400–4000 cm^−1^ at room temperature, using the KBr disk method.

### 2.8 Functional properties

#### 2.8.1 Swelling power and Solubility

Approximately, 1.0 g of starch sample was added to 20 mL of distilled water and heated at 90°C for 30 min with occasional stirring [15]. After heating, the mixture was cooled and centrifuged at 5000 rpm for 10 min. The sediment was weighed, and the supernatant was dried overnight at 105 ± 1 °C.

$$Solubility\;\left(\%\right)=\frac{Dried\;supernatant\;weight\;\left(g\right)}{Sample\;weight\;\left(g\right)}*100$$

$$\mathrm{Swelling}\;\mathrm{power}\;\left(g/g\right)=\frac{\mathrm{Sediment}\;\mathrm{weight}\;(g)}{\mathrm{Weight}\;\mathrm{of}\;\mathrm{dry}\;\mathrm{starc}h\;(g)}$$

#### 2.8.2 Oil absorption capacity

To determine oil absorption capacity (OAC), 0.5 g of starch was mixed with 6 mL of oil in pre-weighed centrifuge tubes. The mixture was incubated for 30 min at 25°C, followed by centrifugation at 5000 rpm for 25 min. The tubes were then inverted over tissue paper for 25 min to remove excess water, and the remaining excess oil was drained. The OAC of starch was calculated using the following formula:

$$\mathrm{OAC}\;\left(g/g\right)=\frac{Weight\;of\;wet\;mass\;\left(g\right)-Weight\;of\;dry\;mass\;\left(g\right)}{\mathrm{Sample}\;weight\;(g)}$$

### 2.9 Bulk density and Tap density

Bulk density was determined using the method outlined by Suriya et al. [16]. Around 3.0 g of starch were placed in a 10 mL measuring cylinder, and the weight per unit volume was recorded.

$$\mathrm{Bulk}\;\mathrm{density}\;\left(g/ml\right)=\frac{\mathrm{Weigh}t\;\mathrm{of}\;\mathrm{the}\;\mathrm{starch}}{\mathrm{Volume}\;\mathrm{of}\;\mathrm{the}\;\mathrm{starch}}$$

Tap density was determined by adding 1.0 g of starch to a graduated cylinder and tapping it mechanically until a constant volume was achieved. Tap density (TD), carr index (CI) and hausner ratio (HR) were calculated using the formulas:

$$\mathrm{Tap}\;\mathrm{density}\;\left(g/ml\right)=\frac{\mathrm{Mass}\;\mathrm{of}\;\mathrm{the}\;\mathrm{starch}}{\mathrm{Final}\;\mathrm{tapped}\;\mathrm{volume}}$$

$$\mathrm{Carr}\;\mathrm{index}=\frac{\mathrm{Tap}\;\mathrm{density}-Bulk\;density}{\mathrm{Tap}\;\mathrm{density}}*100$$

$$\mathrm{Hausner}\;\mathrm{ratio}=\frac{\mathrm{Tap}\;\mathrm{density}}{\mathrm{Bulk}\;\mathrm{density}}$$

### 2.10 Statistical analysis

Statistical analysis was conducted using SPSS software (IBM, Inc., New York) on data obtained from triplicate experiments. One-way ANOVA was performed to analyze significant differences, with Duncan’s multiple range test (DMRT) used for post-hoc analysis (*p*<0.05). Principal component analysis (PCA) was carried out using a correlation matrix derived from the selected data.

## 3 Results and Discussion

### 3.1 Proximate composition of native starches

The proximate analysis of starches extracted from sweet potato (NSP), cassava (NCS) and elephant foot yam (NES), as shown in Table 1**,** revealed significant variations in their composition. The moisture content was highest in NSP (12.33%), followed by NCS (11.06%) and NES (10.01%), respectively. Protein levels were relatively low across all samples, with NSP having the highest protein content (0.79%). Ash content was notably higher in NES (0.66%), indicating the presence of inorganic matter. The amylose content, which influences starch functionality, was highest in NES (22.85%), followed by NSP (21.05%) and NCS (19.28%). Following dual modification, a significant reduction in amylose content was observed in U+A ES-15, compared to U+A SP-9 and U+A CS-3 (Table 2). These results are consistent with findings by Ghoshal and Kaur [17], further confirming the high purity of native starches.

**Table 1:** Proximate analysis of isolated starches from sweet potato, cassava and elephant foot yam starches.*.

Parameters	Starch type**		
	NSP	NCS	NES
Moisture (%)	12.33 ± 0.57^a^	11.06 ± 0.5^b^	10.01 ± 0.10^c^
Ash (%)	0.26 ± 0.11^b^	0.26 ± 0.11^b^	0.66 ± 0.28^a^
Protein (%)	0.79 ± 0.02^a^	0.72 ± 0.04^b^	0.66 ± 0.03^b^
Fat (%)	0.60 ± 0.11^a^	0.40 ± 0.10^b^	0.70 ± 0.10^a^
Total Starch (%)	85.01 ± 0.87^b^	87.40 ± 0.12^a^	84.02 ± 0.12^c^

*Values expressed are mean ± standard deviation. Data of different alphabets in the same row were different with statistical significant (*p* < 0.05).

**NSP, NCS, and NES indicates sweet potato starch, cassava starch, and elephant foot yam starch, respectively.

**Table 2:** Amylose content, relative crystallinity, and functional properties of native and dual modified starches.*.

Samples**	Amylose (%)	RC (%)	Functional properties***		
			**SP (g/g)**	**Solubility (%)**	**OAC (g/g)**
NSP-0	21.05 ± 1.51^a^	21.65 ± 0.07^b^	16.52 ± 0.22^bc^	5.53 ± 0.04^h^	1.26 ± 0.28^a^
U + A SP-3	20.85 ± 0.21^a^	19.58 ± 0.04^e^	16.34 ± 0.15^b^	11.73 ± 0.03^g^	1.24 ± 0.21^a^
U + A SP-9	19.63 ± 1.20^b^	21.58 ± 0.05^b^	17.55 ± 0.26^a^	16.31 ± 0.01^f^	0.93 ± 0.40^a^
U + A SP-15	19.2 ± 1.51^a^	20.58 ± 0.09^d^	16.05 ± 0.02^b^	18.61 ± 0.02^e^	0.82 ± 0.51^a^
NCS-0	19.28 ± 0.67^b^	21.4 ± 0.08^bc^	7.63 ± 0.25^h^	19.63 ± 0.25^d^	1.35 ± 0.26^a^
U + A CS-3	19.02 ± 1.33^a^	19.6 ± 0.20^e^	7.06 ± 0.02^i^	20.46 ± 0.20^c^	1.31 ± 0.43^a^
U + A CS-9	18.84 ± 1.01^ab^	20.65 ± 0.13^d^	7.66 ± 0.27^h^	22.06 ± 0.25^b^	1.38 ± 0.37^a^
U + A CS-15	18.17 ± 0.18^ac^	21.2 ± 0.10^c^	8.55 ± 0.25^g^	24.56 ± 0.20^a^	1.23 ± 0.02^a^
NES-0	22.85 ± 0.14^b^	24.26 ± 0.20^a^	8.82 ± 0.24^g^	4.63 ± 0.20^i^	0.86 ± 0.51^a^
U + A ES-3	22.11 ± 1.70^a^	21.53 ± 0.40^bc^	9.41 ± 0.14^f^	12.53 ± 0.15^g^	1.3 ± 0.44^a^
U + A ES-9	22.06 ± 1.61^bc^	24.44 ± 0.34^a^	10.46 ± 0.13^e^	10.46 ± 0.25^h^	1.28 ± 0.42^a^
U + A ES-15	20.59 ± 0.19^a^	24.53 ± 0.04^a^	11.74 ± 0.09^d^	18.63 ± 0.36^e^	1.28 ± 0.21^a^

*Values expressed are mean ± standard deviation. Data of different alphabets in the same column were different with statistical significant (*p* < 0.05).

**NSP-0- Native sweet potato starch; U + A SP-3,9 &15- ultrasound acetylation sweet potato starch; NCS-0- native cassava starch; U + A CS-3,9 &15- ultrasound acetylation cassava starch; NES-0- native elephant foot yam starch; U + A ES-3,9 &15- ultrasound acetylation elephant foot yam starch.

***RC, SP, and OAC indicates relative crystallinity, swelling power, and oil absorption capacity, respectively.

### 3.2 Morphological characteristics

**Fig. 1:**
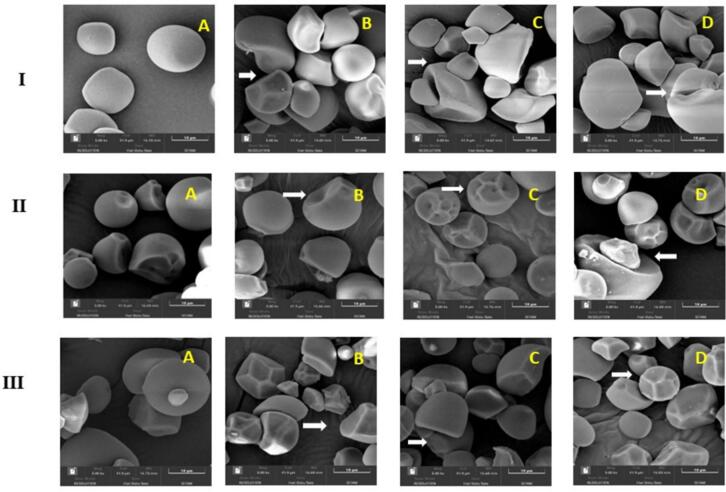
Scanning electron micrographs (SEM) of native and dual modified starches of different sources. (IA: NSP-0, IB: U + A SP-3, IC: U + A SP-9, ID: U + A SP-15; IIA: NCS-0, IIB: U + A CS-3, IIC: U + A CS-9,IID: U + A CS-15 and IIIA: NES-0, IIIB: U + A ES-3, IIIC: U + A ES-9, IIID: U + A ES-15).

The morphology of native and ultrasound-assisted acetylated starch granules is shown in Fig. 1. The SEM images of starches isolated from non-conventional sources, such as sweet potato, cassava and elephant foot yam reveal variations in shape and surface characteristics. Native starches appeared smooth, round, polygonal, and irregular (Fig. 1**: IA, IIA, IIIA)**. As shown in Fig. 1, modified starches exhibited agglomeration and dents with increased treatment time, likely due to acetylation, which introduces hydrophilic groups, enhancing hydrogen bonding and promoting granule fusion [18]. The fragmentation observed in Fig. 1**: IC,** with longer ultrasonication times suggests increased amorphous region, making the starch granule more susceptible to breakdown. This could be useful in applications requiring fast dissolution or modified-release characteristics [19]. The integrity of NCS-0 and NES-0 was less affected by cavitation bubbles compared to NSP-0. The surface roughness of NSP increased with treatment time (Fig. 1**: IC & ID)**. Longer treatment times caused more damage to NSP and NCS, with similar changes observed in NES. These findings align with Abedi et al. [18], who reported that structural changes in potato starches facilitated acetic acid penetration into the granules.

### 3.3 Degree of crystallinity

**Fig. 2:**
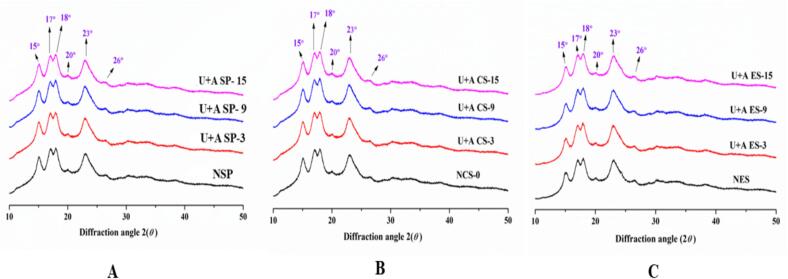
XRD images of native and dual modified starches. (A) sweet potato (B) cassava and (C) elephant foot yam starches.

As shown in Fig. 2**,** XRD analysis provided insights into the crystalline structure of starch granules. Starches with semi-crystalline structures are classified into three types—A, B, and C—based on their distinct XRD patterns. The XRD patterns of sweet potato (NSP) and elephant foot yam (NES) starches exhibited major peaks (2*θ*) at around 15°, 17°, 18°, and 23°, characteristic of C-type starches. In contrast, cassava (NCS) starch exhibited a distinct A-type starch pattern with a single peak (2*θ*) at around 15.01°, and 23.0°, and along with a doublet (2*θ*) at 17.1°, and 18.0°. Following ultrasound-assisted acetylation treatments, the crystalline structures of all isolated starches remained largely unchanged, suggesting that these modification treatments had a minimal impact on the crystallinity of the starch granules. Wang et al. [19] reported no changes in the crystalline region of maize starches after ultrasonication, supporting the information that ultrasonic treatments have minimal impact on crystalline patterns. This is consistent with the findings of the present study, where slight changes in the relative crystallinity (RC) were observed in NSP and NCS after modification, but no observable changes were noted in NES, as shown in Table 2. These results align with previous research indicating that esterification processes primarily occur within the amorphous regions of starch, without significantly altering its crystalline structure [20]. The changes observed in peak intensities might be due to conformational changes resulting from acetylation, which lead to structural rearrangement and fluctuations in the amylose/amylopectin ratio [21].

### 3.4 Functional group analysis

**Fig. 3:**
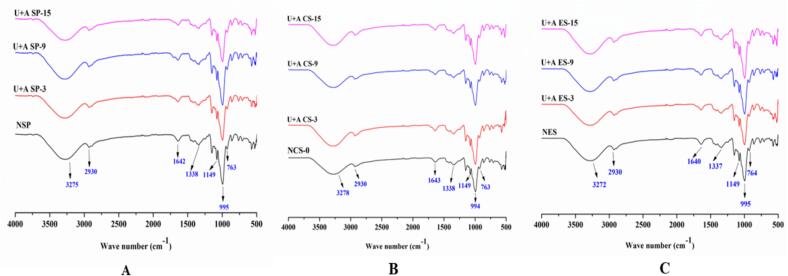
FT-IR images of native and dual modified starches. (A) sweet potato, (B) cassava and (C) elephant foot yam starches.

Fourier-transform infrared (FTIR) spectroscopy was employed to confirm the presence of functional groups, and identify characteristic vibrational bands associated with the folding of amylose and amylopectin [22]. The FTIR spectra for native and dual-modified starches is presented in Fig. 3. The FTIR spectra of both native and dual-modified starches displayed peaks at around wave numbers of 763, 996, 1077, 1149, 1245, 1338, 1614, 2931, and 3280 cm^−1^, confirming the presence of polysaccharides. A broad band observed between 3270 and 3290 cm^−1^ corresponds to complex vibrational stretching involving free inter- and intra-molecular hydroxyl groups, indicating the presence of hydroxyl functionalities [10]. The peak at 2929–2931 cm^−1^ is attributed to C-H stretching from glucose units, associated with the pyransoe ring carbons. Shifts in this region suggest structural changes in amylose or amylopectin due to the modification process. Post-ultrasonication acetylation likely displaced many acetyl groups from the starch chains, as no distinct peak was observed at 1730 cm^−1^, which is typically associated with ester carbonyl stretching. The absorbance peak at 1638–1645 cm^−1^ is attributed to the bending vibration of –OH groups bound to water molecules within the amorphous regions of the starch, while the peak at 1336–1338 cm^−1^ is related to CH_2_ twisting vibrations. Sharp peaks at 1146 cm^−1^ and 1077 cm^−1^ correspond to the coupling of C-O, C-C stretching, and C-O-H bending vibrations within the starch structure. Also, the peak at 928 cm^−^1 is indicative of water sensitivity, a characteristic feature of starch’s hydrophilic nature. Peaks between 763 and 996 cm^−^1 are attributed to hydrogen bonding of –OH groups, skeletal vibrations of α-1,4 glycosidic linkages, and C-C stretching.

### 3.5 Functional properties

#### 3.5.1 Swelling power and solubility

Swelling power and solubility of starch granules are influenced by different factors, including gelatinization temperature, amylose/amylopectin ratio, crystallinity, intermolecular bonds, and the aggregation structure of starch granules [14]. The swelling power of native and modified starches is presented in Table 2. The U+A SP-9 exhibited the highest swelling power (17.55 g/g), suggesting a strong thickening potential. This finding is consistent with the results reported by Singh et al. [14], where ultrasound modification was shown enhance water absorption [12]. In contrast, NCS-0, with the lowest swelling power (7.63), demonstrated limited water retention, in line with the study by Huang et al. [23], which suggested that native cassava starch had lower swelling due to a more rigid granule structure. Similarly, NCS-0 displayed the lowest swelling power, indicting reduced water retention and thickening potential.

The solubility of native and modified starches is presented in Table 2. Results demonstrated that the solubility increased progressively across all dual-modified starches (U+A), showing a significant rise in solubility, likely due to the disruption of molecular structures and the formation of more soluble fragments after dual modification. This trend aligns with observations made by Wang et al. [19], who noted increased solubility in modified starches. Solubility values ranged from 4.63% in NSP-0 to 24.56% in U+A CS-15. The U+A CS-15 exhibited significantly higher solubility, implying a high proportion of easily dissolved components compared to the other samples.

#### 3.5.2 Oil absorption capacity

Oil absorption capacity (OAC) refers to the ability of starch to bind with fat, influencing texture and flavor retention in food products. Table 2 shows the OAC of native and modified starches. As shown in Table 2, NCS-0 exhibited the highest OAC (1.35 g/g), suggesting enhanced emulsifying properties, which makes it suitable for food formulations that require a rich mouthfeel (Table 2). Adebowale et al. [24] reported high OAC in native cassava starch due to its porous structure. In contrast, U+A SP-15 demonstrated the lowest OAC (0.82 g/g), indicating reduced fat-binding capacity, which may limit its effectiveness in improving texture in food products. Studies on dual-modified starches have shown that these starches typically exhibit decreased OAC due to structural changes that reduce the availability of fat-binding sites.

#### 3.5.3 Bulk density and tapped density

Table 3 presents the bulk density (BD) and tapped density (TD) of native and modified starches. As indicateed, U+A CS-3 exhibited a significant increase in BD (0.51 g/ml) compared to NCS-0 (0.36 g/ml), which aligns with research suggesting that ultrasound can densify starch granules by collapsing their internal pores. On the other hand, the BD and TD of NES remained unaffected by the modification, suggesting that ultrasound-acetylation had a minimal impact, likely due to intrinsic of the granules. The Carr index (CI) and Hausner ratio (HR) are indicators of flowability, with lower values reflecting better flow. U+A CS-3 showed a CI of 17.72, which is higher than that of NCS-0 (Table 3**)**. This reflects that the ultrasound-acetylation introduces roughness and irregularities to the starch surface, affecting its flow. Similarly, NSP samples exhibited a gradual increase in CI with modification, consistent with trends observed in modified NSP starches, where surface roughness influences flow properties.

**Table 3:** Techno-functional properties of native and dual modified starches.*.

Samples**	Parameters***			
	**BD (g/ml)**	**TD (g/ml)**	**CI**	**HR**
NSP-0	0.44 ± 0.02^cd^	0.58 ± 0.01^de^	24.22 ± 0.07^g^	1.32 ± 0.03^d^
U + A SP-3	0.43 ± 0.01^d^	0.42 ± 0.01^f^	36.59 ± 0.02^b^	1.55 ± 0.01^b^
U + A SP-9	0.47 ± 0.01^bc^	0.44 ± 0.04^f^	32.63 ± 0.91^e^	1.47 ± 0.02^c^
U + A SP-15	0.48 ± 0.02^b^	0.68 ± 0.03^ab^	34.33 ± 0.95^d^	1.53 ± 0.03^b^
NCS-0	0.36 ± 0.01^e^	0.62 ± 0.02^cd^	14.30 ± 0.12^j^	1.16 ± 0.01^f^
U + A CS-3	0.51 ± 0.01^a^	0.54 ± 0.02^e^	17.72 ± 0.26^i^	1.23 ± 0.02^e^
U + A CS-9	0.48 ± 0.01^b^	0.70 ± 0.03^a^	24.22 ± 0.19^g^	1.34 ± 0.01^d^
U + A CS-15	0.47 ± 0.01^bc^	0.64 ± 0.01^bc^	31.79 ± 0.10^f^	1.48 ± 0.02^c^
NES-0	0.35 ± 0.01^ef^	0.54 ± 0.01^e^	20.49 ± 0.08^h^	1.25 ± 0.03^e^
U + A ES-3	0.35 ± 0.02^e^	0.73 ± 0.02^a^	35.23 ± 0.05^c^	1.53 ± 0.03^b^
U + A ES-9	0.35 ± 0.01^ef^	0.7 ± 0.02^a^	35.21 ± 0.04^c^	1.55 ± 0.03^b^
U + A ES-15	0.32 ± 0.02^f^	0.58 ± 0.03^de^	44.84 ± 0.02^a^	1.81 ± 0.03^a^

*Values expressed are mean ± standard deviation. Data of different alphabets in the same column were different with statistical significant (*p* < 0.05).

**NSP-0- Native sweet potato starch; U + A SP-3,9 &15- ultrasound acetylation sweet potato starch; NCS-0- native cassava starch; U + A CS-3,9 &15- ultrasound acetylation cassava starch; NES-0- native elephant foot yam starch; U + A ES-3,9 &15- ultrasound acetylation elephant foot yam starch.

***BD, TD, CI and HR indicates bulk density, tapped density, carr index, and Hausner ratio, respectively.

### 3.6 Principal component analysis (PCA)

**Fig. 4:**
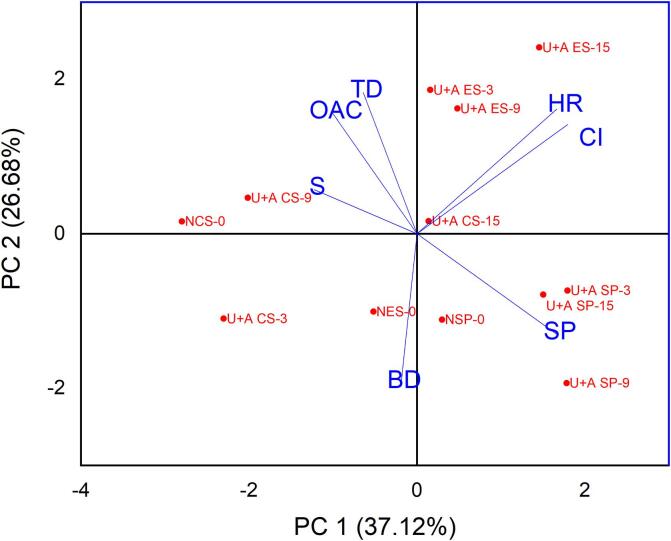
PCA biplots showing relationships between samples (native and dual modified starch granules) and their techno-functional properties.

The PCA biplot (Fig. 4) provides insights into the clustering and relationships of techno-functional properties among native and dual-modification starches derived from NCS, NES, and NSP. The first principal component (PC1) accounts for 37.12% of the total variance, while the second principal component (PC2) represents 26.68%, collectively accounting for a significant portion of the observed variation. The samples are clustered according to their respective modifications, suggesting that dual modifications notably influence the functional properties of starches. Dual-modified NSP starches (U+A SP-3, U+A SP-9, and U+A SP-15) are strongly associated with high values of swelling power. This aligns with previous studies indicating that modifications such as acetylation and ultrasonication enhance water uptake and swelling by improving the accessibility of water to starch granules. In contrast, native starches (NSP-0, NES-0, and NCS-0), which are positioned closer to the origin, display relatively lower values across most functional properties, while ultrasound-acetylated starches (U+A SP, U+A CS, and U+A ES) are distributed across the upper and right quadrant highlighting the influence of modification on functional properties.

The positive correlation of swelling power with modified NSP starches suggests that the structural changes induced by dual modifications enhance their swelling properties. Acetylation, in particular, is known to increase the hydrophilicity and hydration capacity of starches, which is likely responsible for the observed enhancement in swelling power [25]. Additionally, OAC and TD show positive associations with U+A ES-3 and U+A ES-9, indicating that these modifications improve fat-binding capacity, an important feature for emulsion stability [24]. BD and CI exhibit minimal variation across samples, clustering near the origin, suggesting that dual modifications have a limited effect on these properties. Finally, HR are closely linked to U+A CS-15, signifying that dual modifications enhance the solubility of NCS. Overall, the PCA biplot confirms that the dual modification significantly altered the functional properties of non-conventional starches.

## 4 Conclusion

This study explains that dual modification, combining ultrasonication and acetylation, significantly alters the morphological characteristics as well as the physicochemical and techno-functional characteristics of NSP, NCS and NES. Prolonged ultrasonication led to the formation of pores and cracks on the granule surfaces, resulting in agglomeration. XRD and FTIR spectroscopy analyses indicated partial destruction of the crystalline and ordered molecular structures. The dual modification (U+A) notably enhanced properties such as swelling power, and oil absorption capacity, while also affecting tap density and structural integrity. As a result, U+A-modified starches show promise for use as thickening agents and emulsifiers in fast dispersion applications, as well as in the production of edible films. These findings provide valuable insights into the potential applications of dual-modified starches in the food industry. Future research should explore the effects of dual modification on other botanical starch sources and assess their stability and performance in real food systems.

## CRediT authorship contribution statement

**Likhitha Yadav Prakruthi:** Writing – original draft, Methodology, Investigation, Formal analysis, Data curation, Conceptualization. **Hari Krishnan:** Methodology, Data curation. **Tamma Medha:** Writing – review & editing, Validation, Software. **K. Kumarakuru:** Software, Validation, Visualization. **P. Vasantha Kumari:** Software, Validation. **Challa Surekha:** Formal analysis, Resources. **Hemasundar Alavilli:** Formal analysis, Resources. **Deepika Kaushik:** Validation, Data curation. **Abeer Hashem:** Writing – review & editing, Funding acquisition. **Nouf H. Alotaibi:** Writing – review & editing. **Graciela Dolores Avila-Quezada:** Writing – review & editing. **Elsayed Fathi Abd_Allah:** Writing – review & editing, Funding acquisition. **Mukul Kumar:** Writing – review & editing, Validation. **Chagam Koteswara Reddy:** Writing – review & editing, Supervision, Resources, Project administration, Conceptualization.

## Declaration of competing interest

The authors declare that they have no known competing financial interests or personal relationships that could have appeared to influence the work reported in this paper.
